# Associations of Food Insecurity and Memory Function Among Middle to Older–Aged Adults in the Health and Retirement Study

**DOI:** 10.1001/jamanetworkopen.2023.21474

**Published:** 2023-07-03

**Authors:** Peiyi Lu, Katrina Kezios, Neal Jawadekar, Samuel Swift, Anusha Vable, Adina Zeki Al Hazzouri

**Affiliations:** 1 Department of Epidemiology, Mailman School of Public Health, Columbia University, New York, New York; 2 Department of Health Sciences, University of New Mexico, Albuquerque; 3 Department of Family and Community Medicine, University of California, San Francisco

## Abstract

**Importance:**

Food insecurity is a leading public health issue in the US. Research on food insecurity and cognitive aging is scarce, and is mostly cross-sectional. Food insecurity status and cognition both can change over the life course, but their longitudinal relationship remains unexplored.

**Objective:**

To examine the longitudinal association between food insecurity and changes in memory function during a period of 18 years among middle to older–aged adults in the US.

**Design, Setting, and Participants:**

The Health and Retirement Study is an ongoing population-based cohort study of individuals aged 50 years or older. Participants with nonmissing information on their food insecurity in 1998 who contributed information on memory function at least once over the study period (1998-2016) were included. To account for time-varying confounding and censoring, marginal structural models were created, using inverse probability weighting. Data analyses were conducted between May 9 and November 30, 2022.

**Main outcomes and Measures:**

In each biennial interview, food insecurity status (yes/no) was assessed by asking respondents whether they had enough money to buy food or ate less than they felt they should. Memory function was a composite score based on self-completed immediate and delayed word recall task of a 10-word list and proxy-assessed validated instruments.

**Results:**

The analytic sample included 12 609 respondents (mean [SD] age, 67.7 [11.0] years, 8146 [64.60%] women, 10 277 [81.51%] non-Hispanic White), including 11 951 food-secure and 658 food-insecure individuals in 1998. Over time, the memory function of the food-secure respondents decreased by 0.045 SD units annually (β for time, −0.045; 95% CI, −0.046 to −0.045 SD units). The memory decline rate was faster among food-insecure respondents than food-secure respondents, although the magnitude of the coefficient was small (β for food insecurity × time, −0.0030; 95% CI, −0.0062 to −0.00018 SD units), which translates to an estimated 0.67 additional (ie, excess) years of memory aging over a 10-year period for food-insecure respondents compared with food-secure respondents.

**Conclusions and Relevance:**

In this cohort study of middle to older–aged individuals, food insecurity was associated with slightly faster memory decline, suggesting possible long-term negative cognitive function outcomes associated with exposure to food insecurity in older age.

## Key Points

**Question:**

Is exposure to food insecurity associated with changes in memory function among middle to older–aged adults in the US?

**Findings:**

In this cohort study of 12 609 participants, compared with individuals not experiencing food insecurity, US middle to older–aged adults who experienced food insecurity exhibited slightly faster memory decline.

**Meaning:**

The study noted a longitudinal association between food insecurity and memory decline, suggesting possible long-term negative cognitive function outcomes of exposure to food insecurity in older age.

## Teaser

This cohort study examines memory function in middle to older age adults with food insecurity.

## Introduction

Food insecurity, defined as the inadequate and unreliable access to food for healthy life, is a leading public health issue in the US.^1^ In 2020, nearly 40 million people nationwide lived in a food-insecure household,^2^ including approximately 5.2 million (6.8% or approximately 1 in 15) adults aged 60 years or older.^3^ Among middle to older–aged adults, research on food insecurity and health is relatively scarce,^4^ although earlier evidence has shown food insecurity to be associated with various physical and mental health outcomes,^4,5,6,7,8,9^ including cognition.^10,11,12,13,14,15,16,17,18,19^ As population aging accelerates, understanding the association between food insecurity and brain health among older adults is a national public health priority.^20^

To date, most research on food insecurity and cognition in middle to older–age adults has examined this association using cross-sectional data and has suggested that food insecurity is associated with poorer global cognition and lower performance on assessments of executive function and memory^10,11,12^; by comparison, very few studies in adults have examined this association longitudinally.^16,17,18,19^ For example, a longitudinal study of Puerto Rican adults aged 49 to 75 years living in Boston, Massachusetts, reported that food insecurity was associated with a significantly faster 2-year decrease in global cognition and executive function, and a smaller and nonsignificant decrease in memory function.^17^ More recently, in a cohort of US adults older than 65 years with 9 years of follow-up, food insufficiency at baseline was associated with a faster annual decrease in global cognition and immediate and delayed recall, but not executive function.^16^ However, in these studies food insecurity was assessed just once at study baseline. In a study in which food insecurity was assessed at 3 different time points, researchers found that persistently food-insecure older residents in Miami, Florida, were at higher risk of cognitive impairment after 2 years of follow-up, although cognitive decline was not examined in this study.^18^ In the only study, to our knowledge, to examine both food insecurity status and cognition longitudinally, community-dwelling Medicare beneficiaries (aged ≥65 years) who were food insecure at any visit during a 7-year follow-up period had a faster decrease in executive function, but not memory, compared with those who were always food secure.^19^

**Figure 1.**
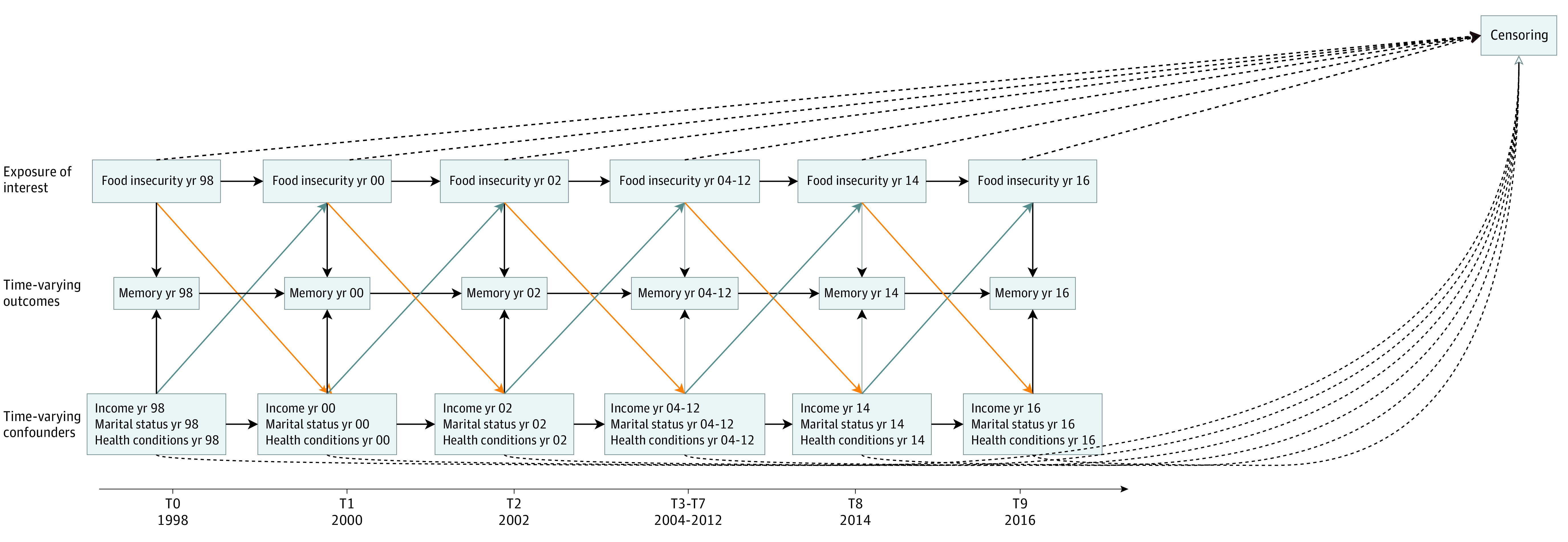
Time-Varying Confounding of Food Insecurity and Memory Function in the Health and Retirement Study Health condition variables included number of chronic conditions, depressive symptoms, body mass index, and current drinking and smoking status. To illustrate the time-varying confounding covariates structure, we provide an example of confounding (blue arrows) and an example of mediation (orange arrows). Dashed lines indicate censoring at each wave. T indicates time point.

When studying the longitudinal association between food insecurity and cognition, time-varying confounding is a concern. As illustrated in Figure 1, food insecurity status and some covariates (eg, income, marital status, and physical health conditions) can vary over time and are likely related to one another. For example, income could influence individuals’ exposure to food insecurity as well as be affected by food insecurity at earlier time points. If this occurs, these covariates act as both confounders (ie, affecting both food insecurity and cognition) and mediators (ie, existing on the pathway from food insecurity to cognition) in the analysis. In this case, standard approaches to confounder control will overadjust for these factors and may result in a biased estimate of the association of food insecurity with cognition.^21,22,23^ To circumvent this issue, marginal structural models (MSMs) with inverse probability weighting can be used to appropriately account for time-varying confounding and, under certain assumptions, provide unbiased estimates of interest. However, these methods have seen limited application in the literature on food insecurity and cognition.

In this study, we build on existing literature by examining the longitudinal association between food insecurity and cognition, using methods that allow for stronger causal inference. In a sample of 12 609 respondents from the Health and Retirement Study (HRS), a national longitudinal cohort of US residents aged 50 years or older, we examined the longitudinal association between food insecurity and memory function during 18 years of follow-up (from 1998 to 2016), using MSMs incorporating inverse probability weights to account for time-varying confounding.^21,22,23,24,25^

## Methods

### Data Source and Analytical Sample

We used data from the HRS, an ongoing national population-based cohort study of Americans aged 50 years and older.^26^ The HRS was launched in 1992 and collects data pertaining to a wide range of topics, such as demographic, health, income, and employment/retirement information. Follow-up surveys are conducted every 2 years by researchers at the University of Michigan. To maintain sample size and representativeness, refresher cohorts are added every 6 years (ie, 1998, 2004, 2010, 2016, and 2020). This study used both core survey data and a user-friendly longitudinal HRS data set prepared by the RAND Corporation,^27^ which are both completely deidentified public data sets; this study did not involve any human participants; therefore, per the Common Rule (45 CFR §46), institutional review board review and approval was not required. The Strengthening the Reporting of Observational Studies in Epidemiology (STROBE) reporting guideline for cohort studies was followed in this study.

We used 1998 as our baseline year, following up participants biennially through examination years 2000, 2002, 2004, 2006, 2008, 2010, 2012, 2014, and 2016. To be included in our sample, respondents had to be aged 50 years or older in 1998 (N = 17 802). Among them, we further excluded respondents missing information on food insecurity status in 1998 (n = 4876) as well as those lacking information on memory function at any visits between 1998 and 2016 (n = 59), resulting in a sample size of 12 867. Data analyses were conducted between May 9 and November 30, 2022.

### Assessment of Food Insecurity Status

Consistent with earlier research,^28,29,30^ we used 2 items to assess food insecurity status. Respondents were first asked if they “have always had enough money to buy the food they need” since the last interview with the response options: yes, no, don’t know, or refused. For those who did not give an affirmative answer to the first question, they were then asked if they “ever ate less than they felt they should because there wasn’t enough money to buy food” in the past 12 months. Individuals who answered no to the first question or yes to the second question were defined as food insecure; otherwise, they were considered food secure. Food insecurity was repeatedly assessed using these questions at every HRS wave between 1998 and 2016.

### Assessment of Memory Function

In the HRS core interviews, cognitive functioning is assessed across 2 domains: memory and mental status. All respondents (aged ≥50 years) complete memory assessments, and assessments of mental status are completed only by respondents aged 65 years and older. To avoid a substantial loss in sample size and retain middle-aged adults in our sample, we focused this analysis on memory function, using a previously validated composite score.^31,32,33,34,35^ In brief, HRS respondents’ memory is assessed directly via immediate and delayed word recall tasks. For respondents unable to complete the cognitive tasks independently (ie, those with cognitive impairment), proxy informants (typically their spouses or family members) provide assessments of their cognitive function using validated instruments (ie, a 5-point Likert scale for memory performance and a 16-item version of the Informant Questionnaire for Cognitive Decline). By using the full neuropsychological tests and dementia diagnoses from the Aging, Demographics, and Memory Study, a subsample of HRS respondents, Wu and colleagues^31^ developed an approach to estimate a continuous memory function score combining information from both proxy and direct memory assessments at each survey year starting from 1995. At each survey year, this composite memory score is standardized using the 1995 mean (SD). For this analysis, we used memory scores measured between 1998 and 2016; across all survey years, the observed scores in our sample ranged from −2.30 to 2.57, where a higher score indicates better memory function.

### Assessment of Covariates

Covariates of interest included time-invariant baseline confounders measured in 1998 and time-varying confounders that were recorded at each wave and time-updated between 1998 and 2016. Based on earlier literature,^10,11,12,16,17,18,19,36,37,38,39^ these confounders were hypothesized to affect both food insecurity status and cognitive function and decline; we used a directed acyclic graph to specify our assumptions regarding the associations between confounders, food insecurity exposure, and memory over time (Figure 1). As shown in the directed acyclic graph, we posited the existence of exposure-confounder feedback whereby the time-varying confounders are both influenced by exposure to food insecurity at one time point and affect food insecurity status at a later time point. This means that these time-varying covariates can act both as confounders influencing both food insecurity and memory at time (*t*) and mediators (existing on the pathway from food insecurity at *t* *−* *1* to memory at *t*).

The following covariates were considered, with the first being baseline (ie, 1998) time-invariant covariates: age (in years, continuous), sex (female vs male), educational level (in years, continuous), self-reported race and ethnicity (non-Hispanic White vs other [collapsed because the cell size in each subgroup was small]), highest parental level of education (missing, high school or less vs more than high school), and born in southern states (yes vs no) classified by the US Census region, including Alabama, Arkansas, Delaware, Florida, Georgia, Kentucky, Louisiana, Maryland, Mississippi, North Carolina, Oklahoma, South Carolina, Tennessee, Texas, Virginia, and West Virginia. Race and ethnicity was included, with each an important confounder for food insecurity and memory.

The second group was time-varying demographic covariates, including marital status (married/partnered vs other), household income (continuous), and household wealth (continuous). The third group was time-varying health covariates including the number of chronic conditions, which is the sum of 8 medical conditions, such as hypertension and diabetes (range, 0-8); depressive symptoms as assessed by the 8-item Center for Epidemiologic Studies–Depression scale (range, 0-8, a higher value indicating more symptoms); self-reported body mass index, calculated as weight in kilograms divided by height in meters squared (continuous); and current drinking and smoking status (yes/no). Household income and wealth were divided by the square root of household size to account for different household size and composition and then log-transformed to stabilize variance. The last observation carried forward method was used to handle missing data for exposure and for covariates between survey waves for participants who remained alive and under observation (including those who did not respond at a given time point). eTable 1 in Supplement 1 presents the proportion of missing exposure and covariate data among the respondents who remained alive and under observation over time before carrying forward imputation.

### Statistical Analysis

We first described respondents’ characteristics overall and across food insecurity status at baseline (1998). We also examined the overall proportion of respondents who were lost from the study or died over the course of follow-up, as well as by food insecurity status at baseline (eTable 2 in Supplement 1 provides the percentage of censored and active participants at each study visit and stratified by food insecurity status at baseline).

We then used an MSM with inverse probability of treatment (ie, exposure) weights to estimate whether there was an association between food insecurity status and memory decline while accounting for the time-varying confounding structure (Figure 1).^23,40^ Using standard methods (eg, regression) in the presence of exposure-confounder feedback would be inappropriate,^41^ because regression adjustment removes the portion of the exposure that works through (ie, is mediated by) the time-varying confounders and could also induce collider-stratification bias.^42^ Marginal structural models are able to overcome this challenge through weighting; that is, adjustment for time-varying confounders is achieved by using them as predictors in the construction of inverse probability of exposure weights such that in the weighted sample the association between the time-varying confounders and exposure at time *t* is removed but the association between exposure at *t* *−* *1 *and the outcome that works through that confounder remains.^21,41^ Under the assumptions of conditional exchangeability, positivity, and no model misspecification,^21,22,23^ the MSM will produce unbiased effect estimates.

To compute inverse probability of treatment (ie, exposure) weights (IPTWs), for each visit (equation 1), we ran a logistic regression model predicting the probability (ie, propensity score) of food insecurity status (A) at time *t* conditional on all time-invariant (V) and time-varying covariates (L) measured at *t* *−* *1 *listed previously, including food insecurity at the previous visit (*t-1*). The predicted probability of food insecurity constituted the denominator of the weight. To stabilize the weight, we included as a numerator the predicted probability of food insecurity at time *t* conditional on food insecurity at the previous visit (*t* *−* *1*). We repeated this process and generated an IPTW for each person at each study visit.



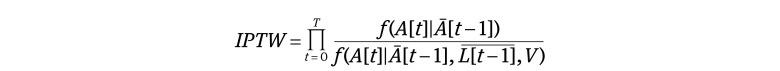



To account for potential selective attrition due to participants dropping out of the sample over time, we used the same procedure as above to compute the inverse probability of censoring weights (IPCWs) for each person at each follow-up visit.^43,44^ We used the same covariates from the IPTW models to compute IPCWs, positing that major predictors of attrition overlapped with our set of predictors of exposure to food insecurity (equation 2); C represents censoring status, where C = 0 is uncensored).



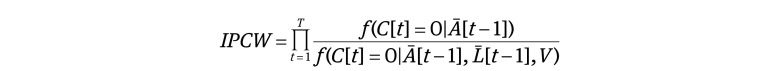



Because most respondents in this study were of older age at baseline (mean [SD], 67.7 [10.95] years), we believed it was not reasonable to include death in our definition of censoring, which would create a pseudopopulation in which no one died during the study period of 18 years.^45^ Furthermore, because mortality is a competing event over the course of follow-up, when a respondent dies this prevents any further memory decline from occurring, making their outcomes after truncation by death undefined.^40^ To our knowledge, there is not a consensus approach for dealing with censoring by death in this situation.^45,46^ Therefore, in this study, censoring only represents being lost to follow-up from causes other than death.

We created plots of the estimated probability density function of the propensity score for food insecurity and the propensity score for censoring for each time point of exposure from 1998 to 2016 to check for potential violations of the positivity assumption. We similarly assessed covariate balance at each time point by plotting the standardized mean difference in measured confounders in the sample before and after reweighting. The final inverse probability weight at each time point was the cumulative multiplication product of IPTWs and IPCWs per person across visits up to time *t*. To avoid extreme weights, we truncated the weights at the 1st and 99th percentiles,^23^ resulting in weights with a mean (SD) across all time points of 0.98 (0.31). With weight truncation, the final analytic sample size became 12 609.

In addition, we used a linear mixed-effects model weighted by the final time-updated inverse probability weights to examine the association between food insecurity and memory decline between 1998 and 2016. We used years since baseline as our time scale (adjusting for baseline age in the construction of the weights). We modeled time as a linear term to maintain interpretability of the estimates and because the addition of a quadratic term for time did not improve model fit. The linear mixed-effects model allows for individual random effects and has 3 coefficients of interest. The coefficient for food insecurity (reference: food secure) estimates the association of food insecurity with baseline memory function. The coefficient for time estimates the yearly rate of change in memory function among food-secure respondents, where a negative coefficient indicates memory decline over time. The interaction coefficient for food insecurity × time is the main coefficient of interest and it estimates the association between food insecurity and rate of memory change over time. Bootstrapped 95% CIs were calculated from 1000 bootstrap samples.

To facilitate interpretation, we translated the food insecurity × time estimates into a measure of excess years of memory aging over a 10-year period, as has been done in earlier research.^44^ To do this, we divided the difference in the yearly rates of memory decline between food-insecure and food-secure respondents (ie, the coefficient of interaction term food insecurity × time) by the yearly rate of memory decline for the food-secure respondents (ie, the coefficient for time, assumed to represent baseline aging), and we multiplied this value by 10. A positive value means food-insecure individuals had more (ie, excess) memory aging during a 10-year period compared with food-secure individuals. All analyses were conducted in R, version 4.2.1 (R Foundation for Statistical Computing),^47^ using packages dplyr^48^ and lme4.^49,50^

Two sensitivity analyses were conducted to address concerns about residual confounding. First, to better control for differences in early-life social conditions, we replaced parental educational attainment in our models with a previously validated measure of early-life socioeconomic circumstances that incorporates dimensions of human, social, and financial capital.^51^ Second, given only a 2-year interval between survey waves, we were concerned that treating health conditions as time-varying covariates may have overadjusted for this confounder (that is, while 2 years may be a reasonable time for food insecurity status at one time point to impact a respondent’s income or body mass index at the next time point, it may be too short to be the cause of a new diagnosis of a chronic health condition); therefore, we reran models for IPTWs treating health conditions as a time-invariant confounder (while still allowing it to be time-varying in IPCW models) and recomputed estimates from the MSM. We also conducted several sensitivity analyses to evaluate whether different methods for dealing with missing exposure data changed our findings. Specifically, instead of carrying forward exposure values from earlier time points (among respondents alive but did not respond at a particular survey year), we examined a scenario in which all missing exposure data across time points was recoded as food secure and another scenario in which it was recoded as food insecure. We also conducted a scenario with a complete case analysis. Finally, we examined characteristics of participants who (1) remained alive by the end of follow-up, (2) dropped out of the study before the end of follow-up, and (3) died before the end of follow-up, to assess the potential outcomes of mortality-related attrition. Significance testing was unpaired and 2-sided.

## Results

The analytic sample included 12 609 respondents, including 11 951 (94.8%) food-secure and 658 (5.2%) food-insecure individuals in 1998. At baseline, the mean (SD) age was 67.71 (10.95) years, 8146 (64.60%) individuals were women, and 4463 (35.40%) were men. Most respondents were non-Hispanic White (81.51%), attained a mean (SD) of 13.01 (2.98) years of education, and 6439 (51.07%) were married (Table 1). Compared with food-secure respondents at baseline, those who experienced food insecurity were younger and were less likely to be men, be non-Hispanic White, be married, or have parents with more than a high school level education; food-insecure respondents were also more likely to be born in the South and smoke, reported more chronic conditions and depressive symptoms, attained fewer years of education, and had higher body mass index and lower household income and wealth (Table 1).

**Table 1.** Sample Characteristics at Baseline (1998) Overall and Stratified by Baseline Food Insecurity Status, Health and Retirement Study

Characteristic	Overall (n = 12 609)	Food secure (n = 11 951)	Food insecure (n = 658)
Age, mean (SD), y	67.71 (10.95)	67.80 (10.98)	66.07 (10.41)
Sex, No. (%)			
Male	4463 (35.40)	4277 (35.79)	186 (28.27)
Female	8146 (64.60)	7674 (64.21)	472 (71.73)
Non-Hispanic White, No. (%)^a^	10 277 (81.51)	9884 (82.70)	393 (59.73)
Education, mean (SD), y	13.01 (2.98)	13.09 (2.92)	11.50 (3.53)
Married, No. (%)	6439 (51.07)	6213 (51.99)	226 (34.35)
Parental education >HS, No. (%)	1429 (11.33)	1378 (11.53)	51 (7.75)
Born in South, No. (%)	4726 (37.48)	4382 (36.67)	344 (52.28)
Income (per $1000), median (IQR)^b^	20.46 (10.78-38.40)	21.24 (11.49-39.60)	8.48 (5.65-17.32)
Wealth (per $1000), median (IQR)^b^	86.27 (24.00-218.14)	91.22 (28.00-227.20)	12.67 (0.14-55.38)
Chronic conditions, median (IQR)	1 (1-2)	1 (1-2)	2 (1-3)
Depressive symptoms, median (IQR)	1 (0-3)	1 (0-2)	2 (1-5)
BMI, mean (SD)	26.66 (5.34)	26.57 (5.24)	28.31 (6.73)
Current drinking, No. (%)	5823 (46.18)	5610 (46.94)	213 (32.37)
Current smoker, No. (%)	2101 (16.66)	1946 (16.28)	155 (23.56)
Memory function, mean (SD)	0.94 (0.67)	0.94 (0.66)	0.88 (0.70)

Abbreviations: BMI, body mass index (calculated as weight in kilograms divided by height in meters squared); HS, high school.

^a^ Race and ethnicity were self-reported by participants. The other race and ethnicity category included respondents who reported being non-Hispanic and Black, Hispanic, and non-Hispanic and of another racial category; data were collapsed because of small sample sizes.

^b^ Household income and wealth were each divided by the square root of household size.

**Figure 2.**
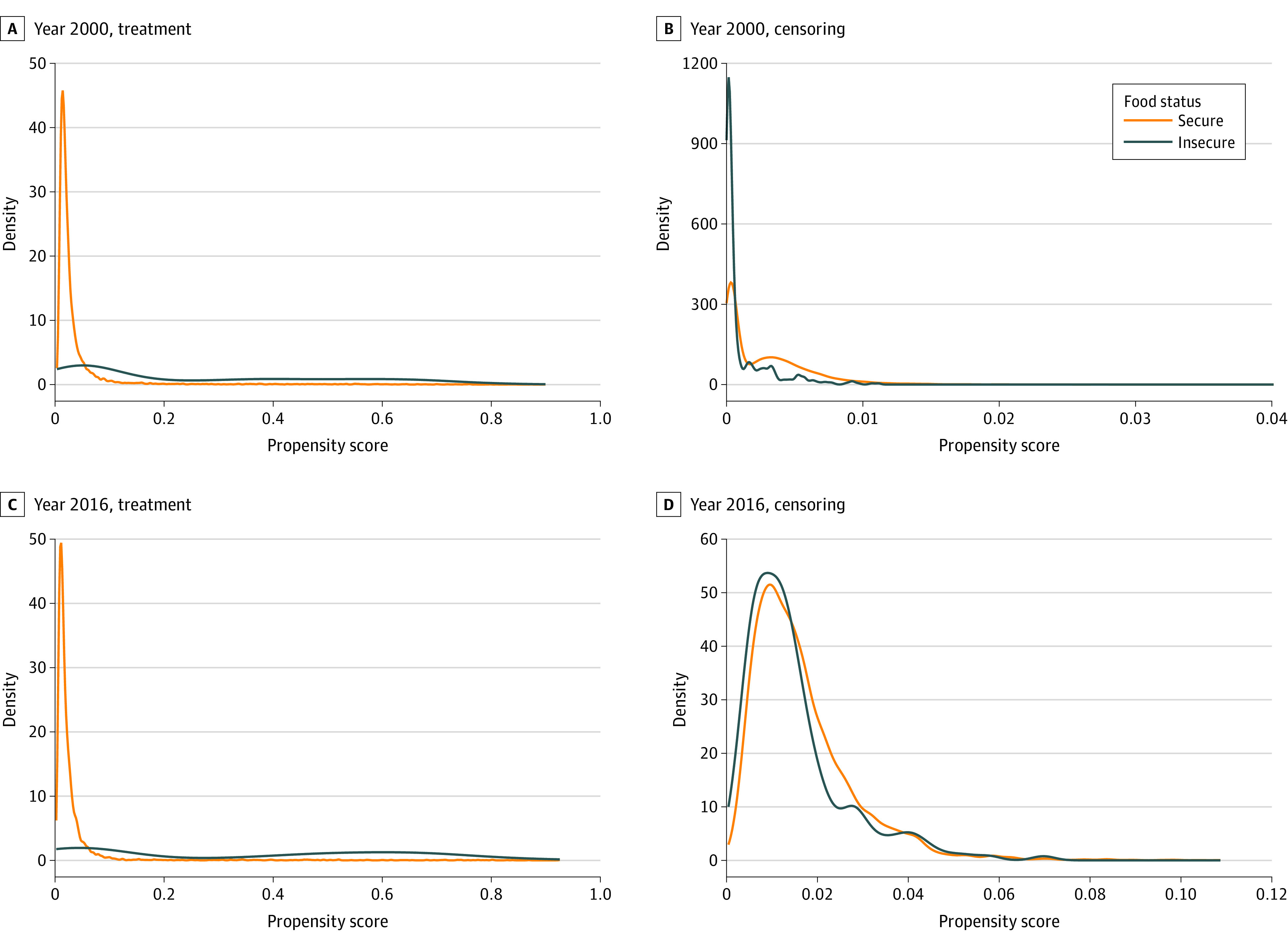
Estimated Probability Density Function of Propensity Score of Treatment (ie, Food Insecurity) and Censoring Among Food-Secure and Food-Insecure Groups in Years 2000 and 2016 of the Health and Retirement Study The propensity score model was adjusted for confounders, including food insecurity at the previous visit, baseline time-invariant demographic characteristics (age, sex, race and ethnicity, years of education, highest level of parental education, if born in Southern states), and other demographic and health characteristics at the previous visit (marital status, household income, household wealth, number of chronic conditions, depressive symptoms, body mass index, and current drinking and smoking status).

Figure 2 plots the estimated probability density function of treatment (ie, food insecurity) and censoring in 2000 and 2016 survey years (as illustrative examples), showing there is sufficient overlap in the propensity score between food-secure and food-insecure groups over time; the probability of being censored from the study increased from 2000 to 2016. The eFigure in Supplement 1 additionally plots (as an illustrative example) the standardized mean differences in measured confounders before and after reweighting the sample by the IPTWs for the 2000 and 2016 survey years, showing that standardized mean differences were within 0.25 after weighting, which suggests good covariate balance.

**Figure 3.**
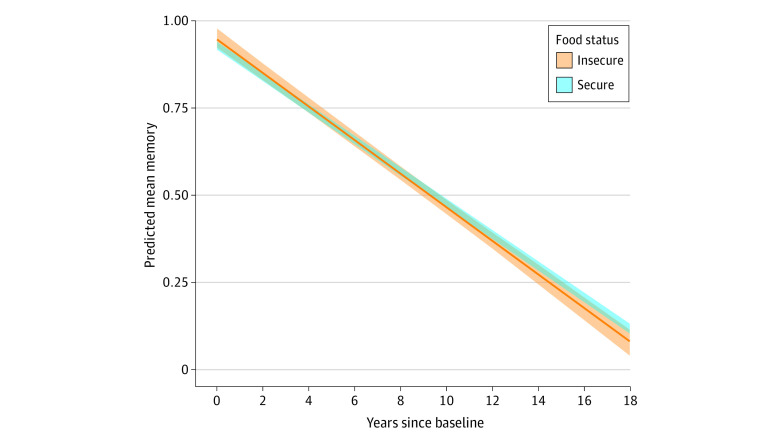
Predicted Memory Function Trajectory for Food-Secure and Food-Insecure Participants, Based on Marginal Structural Models, Health and Retirement Study, 1998-2016 (N = 12 609) The 2 most extreme trajectories in the sample (respondents who are food insecure at all time points and those who are food secure at all time points) are shown.

Table 2 presents the coefficients of interest from the MSM. Food insecurity was not associated with baseline memory function (β for food insecurity, 0.017; 95% CI, −0.014 to 0.046 SD units). Over time, the memory function of the food-secure respondents decreased by 0.045 SD units annually (β for time, −0.045; 95% CI, −0.046 to −0.045 SD units); the significant and negative interaction term between food insecurity status and time suggests that the memory decline rate was faster among food-insecure respondents than food-secure respondents, although the magnitude of the coefficient was small (β for food insecurity × time, −0.0030; 95% CI, −0.0062 to −0.00018 SD units). To aid in interpretation, Figure 3 plots the estimated rate of memory decline comparing being food insecure at every time point and being food secure at every time point. When translated into excess years of memory aging per decade, these estimates suggest that food-insecure respondents may experience an estimated 0.67 additional (ie, excess) years of memory aging during a 10-year period compared with food-secure respondents.

**Table 2.** Associations Between Food Insecurity and Memory Function Using Marginal Structural Models, Health and Retirement Study, 1998-2016^a^

Variable	β (95% CI)	*P* value	Excess years of memory aging per decade^b^
Food insecurity, SD units	0.017 (−0.014 to 0.046)	.17	[Reference]
Time (years since baseline), SD units	−0.045 (−0.046 to −0.045)	<.001	NA
Food insecurity × time^c^	−0.0030 (−0.0062 to −0.00018)	.02	0.67

Abbreviation: NA, not applicable.

^a^ Estimates are from linear mixed effects models that are weighted by the inverse probability of treatment and attrition, as detailed in the Methods section.

^b^ To compute the excess years of memory aging per decade, the coefficient for the interaction term (food insecurity × time) was divided by the yearly rate of memory decline among food-secure respondents (ie, the coefficient for time, which was assumed to represent normal or baseline aging), and this quantity was multiplied by 10. A positive value indicates excess aging for food-insecure respondents.

^c^ The coefficient for food insecurity × time estimates the difference in the rate of memory decline comparing food insecure with food secure respondents over time.

Sensitivity analyses adjusting for childhood socioeconomic conditions rather than just parental educational level (eg, β for food insecurity × time, −0.0029; 95% CI, −0.0059 to 0.000035 SD units) or treating chronic health conditions as a time invariant rather than time-varying confounder in IPTWs (eg, β for food insecurity × time, −0.0029; 95% CI, −0.0061 to 0.000078 SD units) produced similar findings to the main analyses. Conclusions were similar regardless of methods used to impute exposure information except in the scenario in which all imputed exposure information was recoded as food insecure (eg, β for food insecurity × time, 0.00093; 95% CI, −0.00068 to 0.0026 SD units); however, this extreme-case scenario is the least realistic of all examined approaches as it substantially exaggerates the population prevalence of food insecurity in the sample, particularly by the end of follow-up. eTable 3 in Supplement 1 reports that, in general, a higher proportion of respondents who died by the end of follow-up were food insecure at baseline compared with respondents who remained in the study or were lost to follow-up for reasons other than death; respondents who died over the course of follow-up also had lower baseline memory scores and more risk factors for both food insecurity and memory decline (eg, higher mean age, lower educational attainment, and lower income and wealth).

## Discussion

Using longitudinal data spanning 18 years, we examined the association between food insecurity and memory changes, using an MSM to deal with methodologic issues of time-varying confounding and attrition to enable more rigorous assessment of the association between food insecurity and memory function over time. Overall, we observed food insecurity was associated with slightly faster memory decline over follow-up, suggesting possible long-term negative cognitive function outcomes regarding exposure to food insecurity in middle to older–aged individuals.

This finding is consistent with and contributes to the nascent literature examining the association between food insecurity and cognition in middle to older–aged adults; although relatively small in magnitude, the observed association is consistent in direction with results from 3 longitudinal studies linking food insecurity to faster cognitive decline,^16,17,19^ including memory decline.^16,17^ There are several pathways through which this association may develop.^10,11^ Without reliable access to sufficient and nutrient-rich foods, individuals with food insecurity may have difficulty maintaining healthy nutritional intake and high-quality diets.^7,52^ In turn, poor diet quality has been associated with cognitive decline.^53,54,55^ Furthermore, individuals living with food insecurity experience financial vulnerability and persistent worries about access to food, which may contribute to them experiencing higher and chronic levels of psychological distress^6,9^; this food insecurity–related chronic stress may contribute to physiologic erosion of brain structure, for example, by stimulating the release of cortisol and increasing systemic inflammation, and ultimately impairing cognitive functioning.^56,57^ Such pathways through which food insecurity may harm brain health over time and eventually accelerate memory decline in older age should be evaluated.

Considering the large number (5.2 million in 2020) of older Americans living with food insecurity^3^ and the increasing burden of Alzheimer disease and related dementias (5 million in 2014 and projected to double by 2060),^58^ food insecurity in older adults is an important area for both future research and policy advocacy. For example, the Supplemental Nutrition Assistance Program (SNAP) is one of the largest social assistance programs targeting food insecurity in the US. However, despite its reported health benefits,^20,34,59,60^ SNAP is largely underused by eligible older adults (only 40% of eligible older US residents used SNAP in 2017).^61^ Measures such as expanding the eligibility criteria,^62^ simplifying administrative procedures,^62^ and improving benefit levels^4^ may be potential avenues to help improve SNAP participation among this group.^34^ Additionally, studies show food pantry programs, such as food banks and soup kitchens, run by nonprofit organizations can help improve food security,^63,64^ underscoring the importance of governmental and other public efforts to expand financial support for food banks and other emergency food systems.^65^ Research assessing the potential cognitive health benefits of participation in such social programs in older age is warranted.

### Strengths and Limitations

This study has some notable strengths. While existing studies on food insecurity and cognition are typically cross-sectional,^10,11^ this study used longitudinal data over an extended period (ie, 18 years). Our composite memory score outcome incorporated proxy assessments, where applicable, enabling retention of respondents with severe cognitive impairment who would otherwise be excluded from analyses. Furthermore, we used MSM to account for time-varying confounding and censoring, providing a rigorous evaluation of the longitudinal association between food insecurity and memory over time. Our findings may advance the understanding of the association between food insecurity and cognitive changes in middle to older–aged individuals for which there has been limited research to date.

The study has limitations. The interpretation of our estimates as causal relies on satisfaction of 3 key assumptions: conditional exchangeability, positivity, and no model misspecification at each time interval. There were no obvious violations of the positivity assumption on visual inspection. In addition, weight truncation improved the behavior of our final IPTW and ITCW models, and modeling time nonlinearly (ie, including a quadratic time term in the model) did not improve model fit of the MSM, reducing, although not eliminating, concerns about potential model misspecification. However, model misspecification could occur due to unmeasured confounding. Unmeasured confounding, which is empirically unverifiable and difficult to resolve in observational settings, would violate conditional exchangeability and is likely the biggest threat to the validity of our study findings. The magnitude of our main coefficient of interest (β for food insecurity × time) was quite small, and associations of smaller magnitude are more sensitive to unmeasured confounding (meaning even fairly weak unmeasured confounding could explain away these associations). However, our analyses controlled for many key predictors of both food insecurity and cognition, such as years of education, household income, and wealth, and our findings were similar in several sensitivity analyses conducted to address potential residual confounding. Furthermore, it is possible that the estimate in our study is an underestimate because mortality-related attrition was not accounted for. Descriptive analyses suggest that both food insecurity and cognition predicted mortality, and that participants who died over the course of follow-up had more risk factors related to both food insecurity and worse cognition than those either lost to follow-up or remaining under observation. Thus, over time, the respondents contributing to the analyses were likely a relatively healthier sample, which could mute study estimates. In addition, the composite memory score outcome used in this analysis may produce unreliable estimates of memory function for respondents from historically marginalized racial and ethnic groups (in particular, Hispanic individuals),^31,66^ who are also disproportionately burdened by food insecurity, which could bias study estimates in ways difficult to predict. In light of these concerns, caution is warranted in the interpretation of associations as causal.

In addition to these frequently invoked assumptions, consistency is an unverifiable assumption often implicitly assumed to hold in studies using MSMs.^23^ Consistency is more likely to be satisfied when the study exposure can be conceptualized as a well-defined intervention (eg, exposure is an actual treatment). This is not the case with our exposure, food insecurity, which is a multidimensional and complex construct that likely varies across populations with respect to severity, frequency, and types of food available. It would be unreasonable to assume that food insecurity or interventions on food insecurity would have the same associations with memory in other populations, irrespective of these features. As is common in research studies, we are limited by the granularity of the collected data; although we used a validated measure of food insecurity (yes/no), we lacked information on factors such as severity and level of hunger. These dimensions could impact memory function differently, and it is also possible that they could have varying outcomes in different domains of cognition that we were unable to examine in this study. Thus, future research in populations with varying experiences of food insecurity that have perhaps measured food insecurity more comprehensively using tools such as the US Household Food Security Survey Module^67^ should seek to clarify the aspects of food insecurity that most impact cognition (exploring cognitive domains beyond just memory function) and improve understanding of the mechanisms through which this relationship works. This would be an important step toward identifying the most promising routes to alleviate food insecurity and reduce its potential cognition-related health burdens.

## Conclusions

In this cohort study, food insecurity was associated with slightly faster memory decline compared with food security, suggesting that food insecurity could have a long-term negative consequence on older adults’ memory function. Future work should investigate the underlying mechanisms through which food insecurity influences cognitive health and evaluate the potential cognitive health benefits of food assistance programs.

## Notes

Supplement 1. **eFigure.** Standardized Mean Differences in Confounders Between Food-Secure and Food-Insecure Groups Before and After Reweighting by the Propensity Score in Years 2000 and 2016, Health and Retirement Study **eTable 1.** Frequency (Percentage) of Missing Values in Covariates at Each Time Point Among Those Who Remained Alive and Under Observation, HRS, 1998-2016 **eTable 2.** Number (Percentage) of Participants Censored (Lost to Follow-up) and Active at Each Time Point, HRS, 1998-2016 **eTable 3.** Sample Characteristics at Baseline (1998) Among Respondents Who Were Lost From the Sample by the End of Follow-up (2016), Who Died by the End of Follow-up, and Who Remained in the Study for the Duration of Follow-up

Supplement 2.
Data Sharing Statement
