# Supplementary material for: Associations of Food Insecurity and Memory Function Among Middle to Older–Aged Adults in the Health and Retirement Study
Source: JAMA Netw Open. 2023 Jul 3;6(7):e2321474. doi: 10.1001/jamanetworkopen.2023.21474 (PMC10318471; doi:10.1001/jamanetworkopen.2023.21474)
Supplement: Supplement 1. — eFigure. Standardized Mean Differences in Confounders Between Food-Secure and Food-Insecure Groups Before and After Reweighting by the Propensity Score in Years 2000 and 2016, Health and Retirement Study eTable 1. Frequency (Percentage) of Missing Values in Covariates at Each Time Point Among Those Who Remained Alive and Under Observation, HRS, 1998-2016 eTable 2. Number (Percentage) of Participants Censored (Lost to Follow-up) and Active at Each Time Point, HRS, 1998-2016 eTable 3. Sample Characteristics at Baseline (1998) Among Respondents Who Were Lost From the Sample by the End of Follow-up (2016), Who Died by the End of Follow-up, and Who Remained in the Study for the Duration of Follow-up [file jamanetwopen-e2321474-s001.pdf]

Supplementary Online Content

Lu P, Kezios K, Jawadekar N, Swift S, Vable A, Zeki Al Hazzouri A. Associations of food insecurity and memory function among middle to older–aged adults in the Health and Retirement Study. *JAMA Netw Open*. 2023;6(7):e2321474. doi:10.1001/jamanetworkopen.2023.21474

eFigure. Standardized Mean Differences in Confounders Between Food-Secure and Food-Insecure Groups Before and After Reweighting by the Propensity Score in Years 2000 and 2016, Health and Retirement Study

eTable 1. Frequency (Percentage) of Missing Values in Covariates at Each Time Point Among Those Who Remained Alive and Under Observation, HRS, 1998-2016

eTable 2. Number (Percentage) of Participants Censored (Lost to Follow-up) and Active at Each Time Point, HRS, 1998-2016

eTable 3. Sample Characteristics at Baseline (1998) Among Respondents Who Were Lost From the Sample by the End of Follow-up (2016), Who Died by the End of Follow-up, and Who Remained in the Study for the Duration of Follow-up

This supplementary material has been provided by the authors to give readers additional information about their work.

© 2023 Lu P et al. *JAMA Network Open.*

eFigure. Standardized mean differences in confounders between food-secure and food-insecure groups before and after reweighting by the propensity score in years 2000 and 2016, Health and Retirement Study

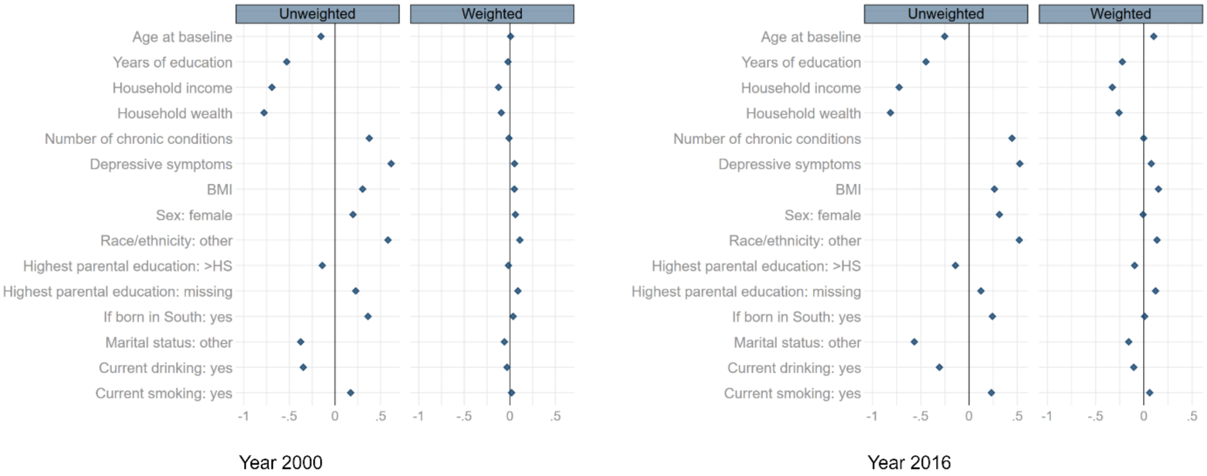

eFigure 1 legend: Unweighted refers to raw data. Weighted refers to inverse probability treatment weights. The reference level was male for sex, non-Hispanic White for race/ethnicity, <=HS for highest parental education, no for born in South, married/partnered for marital status, no for current drinking and smoking.

© 2023 Lu P et al. *JAMA Network Open.*

eTable 1. Frequency (Percentage) of missing values in covariates at each time point among those who remained alive and under observation, HRS, 1998-2016

	Yr 1998	Yr 2000	Yr 2002	Yr 2004	Yr 2006	Yr 2008	Yr 2010	Yr 2012	Yr 2014	Yr 2016
N of alive	12867	11958	10911	10039	9099	8280	7331	6569	5806	5012
Food insecurity	0 (0)	2835 (23.7)	2727 (25.0)	2684 (26.7)	2434 (26.8)	2263 (27.3)	2035 (27.8)	1830 (27.9)	1654 (28.5)	1562 (31.2)
Marital status	17 (0.13)	656 (5.49)	731 (6.70)	838 (8.35)	756 (8.31)	726 (8.77)	708 (9.66)	606 (9.23)	636 (11.0)	752 (15.0)
Household income	0 (0)	641 (5.36)	720 (6.60)	832 (8.29)	756 (8.31)	726 (8.77)	707 (9.64)	600 (9.13)	628 (10.8)	744 (14.8)
Household wealth	0 (0)	641 (5.36)	720 (6.60)	832 (8.29)	756 (8.31)	726 (8.77)	707 (9.64)	600 (9.13)	628 (10.8)	744 (14.8)
Number of chronic conditions	0 (0)	641 (5.36)	720 (6.60)	833 (8.30)	756 (8.31)	726 (8.77)	707 (9.64)	600 (9.13)	628 (10.8)	744 (14.8)
Depressive symptoms	716 (5.56)	1385 (11.6)	1480 (13.6)	1473 (14.7)	1231 (13.5)	1166 (14.1)	1212 (16.5)	1018 (15.5)	989 (17.0)	1057 (21.1)
Body Mass Index	151 (1.17)	791 (6.61)	884 (8.10)	972 (9.68)	879 (9.66)	819 (9.89)	780 (10.6)	659 (10.0)	679 (11.7)	799 (15.9)
Drinking status	2 (0.02)	643 (5.38)	721 (6.61)	832 (8.29)	757 (8.32)	726 (8.77)	708 (9.66)	601 (9.15)	631 (10.9)	750 (15.0)
Smoking status	3 (0.02)	642 (5.37)	720 (6.60)	875 (8.72)	790 (8.68)	759 (9.17)	734 (10.0)	626 (9.53)	652 (11.2)	764 (15.2)

Note: The denominator of percentage of missing data was the number of respondents who remained alive and under observation (including those who did not respond at a given time point).

© 2023 Lu P et al. *JAMA Network Open.*

eTable 2. Number (percentage) of participants censored (Lost to Follow-up) and active at each time point, HRS, 1998-2016

	Yr 1998	Yr 2000	Yr 2002	Yr 2004	Yr 2006	Yr 2008	Yr 2010	Yr 2012	Yr 2014	Yr 2016
**Overall sample, N=12867**										
Active	12867 (100)	11958 (92.94)	10911 (84.80)	10039 (78.02)	9099 (70.72)	8280 (64.35)	7331 (56.98)	6569 (51.05)	5806 (45.12)	5012 (38.95)
Censored	0 (0)	32 (0.25)	83 (0.65)	136 (1.06)	266 (2.07)	304 (2.36)	333 (2.59)	480 (3.73)	600 (4.66)	686 (5.33)
Dead	0 (0)	877 (6.82)	1873 (14.56)	2692 (20.92)	3502 (27.22)	4283 (33.29)	5203 (40.44)	5818 (45.22)	6461 (50.21)	7169 (55.72)
**Food-secure individuals in 1998, N=12062**										
Active	12062 (100)	11217 (92.99)	10236 (84.86)	9414 (78.05)	8528 (70.70)	7760 (64.33)	6888 (57.10)	6180 (51.24)	5465 (45.31)	4718 (39.11)
Censored	0 (0)	31 (0.26)	81 (0.67)	132 (1.09)	258 (2.14)	295 (2.45)	321 (2.66)	461 (3.82)	577 (4.78)	662 (5.49)
Dead	0 (0)	814 (6.75)	1745 (14.47)	2516 (20.86)	3276 (27.16)	4007 (33.22)	4853 (40.23)	5421 (44.94)	6020 (49.91)	6682 (55.40)
**Food-insecure individuals in 1998, N=805**										
Active	805 (100)	741 (92.05)	675 (83.85)	625 (77.64)	571 (70.93)	520 (64.60)	443 (55.03)	389 (48.32)	341 (42.36)	294 (36.52)
Censored	0 (0)	1 (0.12)	2 (0.25)	4 (0.50)	8 (0.99)	9 (1.12)	12 (1.49)	19 (2.36)	23 (2.86)	24 (2.98)
Dead	0 (0)	63 (7.83)	128 (15.90)	176 (21.86)	226 (28.07)	276 (34.29)	350 (43.48)	397 (49.32)	441 (54.78)	487 (60.50)

© 2023 Lu P et al. *JAMA Network Open.*

eTable 3. Sample characteristics at baseline (1998) among respondents who were lost from the sample by the end of follow-up (2016), who died by the end of follow-up, and who remained in the study for the duration of follow-up

	**Participant study status by the end of follow-up (2016)**		
	**Not censored (N=4,875)**	**Lost to follow-up (n=675)**	**Died (n=7,059)**
Age, mean (SD)	60.54 (6.84)	63.45 (8.88)	73.06 (10.42)
Women, n, (%)	3284 (67.36)	426 (63.11)	4436 (62.84)
Non-Hispanic White, n (%)[b]	3977 (81.58)	592 (87.70)	5705 (80.82)
Education, mean (SD)	13.80 (2.50)	13.58 (2.57)	12.40 (3.17)
Married, n (%)	3179 (65.21)	410 (60.74)	2852 (40.40)
Parental education >HS, n (%)	884 (18.13)	116 (17.19)	432 (6.12)
Born in South (%)	1756 (36.02)	222 (32.89)	2746 (38.90)
Income (per $1000), median (Q1, Q3)[c]	28.49 (15.59, 51.23)	29.16 (15.47, 51.89)	15.68 (8.73, 28.31)
Wealth (per $1000),median (Q1, Q3)[c]	111.88 (37.99, 271.52)	123.74 (48.93, 297.54)	66.47 (12.73, 177.54)
Chronic conditions, mean (SD)	1.11 (1.04)	1.28 (1.20)	2.09 (1.40)
Depressive symptoms, mean (SD)	1.32 (1.75)	1.23 (1.69)	1.94 (2.02)
Body mass index (kg/m^2), mean (SD)	27.31 (5.14)	26.48 (4.76)	26.23 (5.48)
Current drinking, n (%)	2691 (55.20)	368 (54.52)	2765 (39.17)
Current smoker, n (%)	725 (14.87)	108 (16.00)	1267 (17.95)
Food insecure, n (%)	220 (4.51)	18 (5.89)	416 (5.89)
Memory function, mean (SD)	1.26 (0.28)	1.18 (0.43)	0.69 (0.76)
**Among participants with memory scores in 1998 and 2000**			
2-year change in memory score (2000-1998), median (Q1, Q3)[d]	-0.043 (-0.17, 0.082)	-0.038 (-0.18, 0.087)	-0.12 (-0.33, 0.062)
% change in memory score (2000-1998), median (Q1, Q3)[d]	-3.41 (-12.61, 6.76)	-2.58 (-13.54, 7.30)	-9.86 (-34.05, 8.41)
**Among participants with at least two memory scores between 2000 and 2002**			
2-year change in memory score (2002-2000), median (Q1, Q3)[e]	-0.033 (-0.16, 0.084)	-0.032 (-0.14, 0.081)	-0.089 (-0.30, 0.075)
% change in memory score (2002-2000), median (Q1, Q3)[e]	-2.63 (-12.91, 7.21)	-2.30 (-11.75, 7.78)	-7.60 (-33.41, 10.51)

Note: SD=standard deviation, IQR=interquartile range, BMI=body mass index, HS=high school, NH=non-Hispanic

© 2023 Lu P et al. *JAMA Network Open.*

[a]All 12609 respondents had complete information at baseline exposure and contributed to at least one time point over follow-up with outcome information. When a respondent died, they ceased contributing to the analysis. When a respondent was lost from the study for reasons other than death, they were coded as censored.
[b]Race and ethnicity were self-reported by participants. The “other race/ethnicity” category included respondents who reported being non-Hispanic and Black, Hispanic, and being non-Hispanic and of another racial category.
[c]Household income and wealth were each divided by the square root of household size.
[d]2-year change in memory score was computed by subtracting scores in 1998 from scores in 2000; percent change was computed by dividing this value by scores in 1998 and multiplying by 100.
[e]To check that 2-year change in memory score was not a function of only examining change between 2000 and 1998, we calculated the 2-year change and % change in memory scores for the years 2002 and 2000 among individuals who contributed scores to both time points. 1998-2000 and 2000-2002 were chosen because they were the time points with the least missing data in memory scores.

© 2023 Lu P et al. *JAMA Network Open.*
