# Supplementary material for: Associations of Food Insecurity and Memory Function Among Middle to Older–Aged Adults in the Health and Retirement Study
Source: JAMA Netw Open. 2023 Jul 3;6(7):e2321474. doi: 10.1001/jamanetworkopen.2023.21474 (PMC10318471; doi:10.1001/jamanetworkopen.2023.21474)
Supplement: Supplement 2. — Data Sharing Statement [file jamanetwopen-e2321474-s002.pdf]

Data Sharing Statement

Lu. Associations of Food Insecurity and Memory Function Among Middle to Older–Aged Adults in the Health and Retirement Study. *JAMA Netw Open*. Published July 03, 2023. doi:10.1001/jamanetworkopen.2023.21474

Data
Data available: Yes
Data types: Deidentified participant data
How to access data: data is publicly available
When available: With publication

Supporting Documents
Document types: None

Additional Information
Who can access the data: anyone can access the data
Types of analyses: any purpose
Mechanisms of data availability: available online
